# Intercellular interactions between mast cells and stromal fibroblasts obtained from canine cutaneous mast cell tumours

**DOI:** 10.1038/s41598-021-03390-w

**Published:** 2021-12-13

**Authors:** Lidia H. Pulz, Yonara G. Cordeiro, Greice C. Huete, Karine G. Cadrobbi, Arina L. Rochetti, Pedro L. P. Xavier, Adriana Tomoko Nishiya, Silvio Henrique de Freitas, Heidge Fukumasu, Ricardo F. Strefezzi

**Affiliations:** 1 Faculdade de Medicina Veterinária e Zootecnia, Universidade de São Paulo, Av. Prof. Dr. Orlando Marques de Paiva, 87, São Paulo, SP CEP 05508-270 Brazil; 2 Laboratório de Oncologia Comparada e Translacional, Faculdade de Zootecnia e Engenharia de Alimentos, Universidade de São Paulo, Campus “Fernando Costa”, Av. Duque de Caxias Norte 225, Pirassununga, SP CEP 13635-900 Brazil; 3 Hospital Veterinário da Universidade Anhembi Morumbi, R. Conselheiro Lafaiete, 64, São Paulo, SP CEP 03101-00 Brazil

**Keywords:** Cancer microenvironment, Cancer, Biomarkers, Oncology, Pathogenesis

## Abstract

Mast cell tumours (MCTs) are the most frequent malignant skin neoplasm in dogs. Due to the difficulty in purifying large numbers of canine neoplastic mast cells, relatively little is known about their properties. A reproducible in vitro model is needed to increase the understanding about the phenotype and functional properties of neoplastic mast cells. In the present study, we describe the establishment of primary cocultures of neoplastic mast cells from canine cutaneous MCTs and cancer-associated fibroblasts. We confirmed the inability of canine neoplastic mast cells to remain viable for long periods in vitro without the addition of growth factors or in vivo passages in mice. Using a transwell system, we observed that mast cell viability was significantly higher when there is cell-to-cell contact in comparison to non-physical contact conditions and that mast cell viability was significantly higher in high-grade than in low-grade derived primary cultures. Moreover, the use of conditioned medium from co-cultured cells led to a significantly higher tumoral mast cell viability when in monoculture. Signalling mechanisms involved in these interactions might be attractive therapeutic targets to block canine MCT progression and deserve more in-depth investigations.

## Introduction

Mast cells play an important role in the pathogenesis of inflammatory and allergic reactions^1^, but neoplastic proliferation can severely affect the dog skin. Mast cell tumours (MCTs) represent 11–27% of all malignant skin neoplasms in this species and the treatment is considered challenging due to the highly variable behaviour of the tumour^2^. Due to the difficulty in reliably purifying large numbers of canine neoplastic mast cells, relatively little is known about their properties. Previous investigations using non-neoplastic mast cells in short-term cultures have contributed to our understanding of phenotypic and functional features^3–7^, but a reproducible in vitro model that employs canine neoplastic mast cells and stromal malignant counterparts should be considered as an effective tool to study the canine mast cell tumour at the cellular and molecular levels.

One of the main reasons for the failure to establish primary cell-cultures from canine MCTs has been the difficulty in isolating and maintaining viable primary cells from the solid neoplastic masses. Most of the studies performed with canine neoplastic mast cell lines in permanent culture were obtained from freshly disaggregated MCTs that were subsequently inoculated in athymic/nude^8–11^ or severe combined immunodeficiency (SCID) mice^12^. Although a great amount of useful data has been generated, the limitation is obvious: malignant mast cells are influenced by the mouse microenvironment. Canine bone marrow-derived mast cells (BMMCs) were also used for in vitro studies^7^ but several important differences were demonstrated between immature BMMCs and mature mast cells through transcriptome analysis^13^, which can limit the relevance of this model.

The process of generating mast cells in vitro is species-specific^7^, suggesting that certain aspects of canine mast cell biology cannot be established with human mast cells. Therefore, in vitro models are needed to reflect MCT biology in vivo more accurately and to enable long-term studies. Human connective tissue mast cells were successfully maintained ex vivo with skin-derived fibroblasts for up to 13 days^14^. Additional coculture studies performed with mouse mast cells and fibroblasts showed that the mast cell phenotype is profoundly influenced by interactions with fibroblast products^15,16^, promoting differentiation in BMMCs^17^, as well as maturation modulated by fibroblasts^18^. On the other hand, mast cells were also able to stimulate stroma modulation through the release of potent fibrogenic substances and induction of matrix metalloproteinases (MMPs) release from fibroblasts when in cell-to-cell contact^19–21^.

Considering the context of solid cancers, several studies have focused on coculture techniques to explore the cancer-mediated regulation by cancer-associated fibroblasts (CAFs) but none of them are exclusive to canine tumours^22–27^. Recently, CAFs were demonstrated to be immersed in MCT stroma^28,29^ but canine neoplastic mast cell interactions with the extracellular microenvironment have not been widely investigated.

In this paper, we describe the establishment of primary co-cultures of neoplastic mast cells and cancer associated fibroblasts (CAFs) from canine cutaneous MCTs and explore the relationship of both cell types by coculture experiments in which cancer cells and fibroblasts were separated by a transwell chamber with micropores that allowed cell-to-cell communication through soluble factors.

## Results

### Study population

Fourteen MCT samples were obtained from twelve dogs immediately after the excisional biopsy. Thirteen were used for cell viability assays and one sample (401–18) was available only for the co-culture assays. The age of the dogs ranged from 3 to 14 years (mean = 7.6 years old). Females (58.3%) were more frequent than males (41.7%). Considering the breed distribution, 41.7% (5/12) of the MCTs cases were mongrel dogs and 58.3% (7/12) were purebred dogs. Seven tumours (50%) were high-grade MCTs and 7 (50%) were low-grade^30^. The characteristics of the dog population and histological classifications of each lesion are described in Table S1 (Supplementary materials).

### Culture characterization of canine MCT

**Figure 1:**
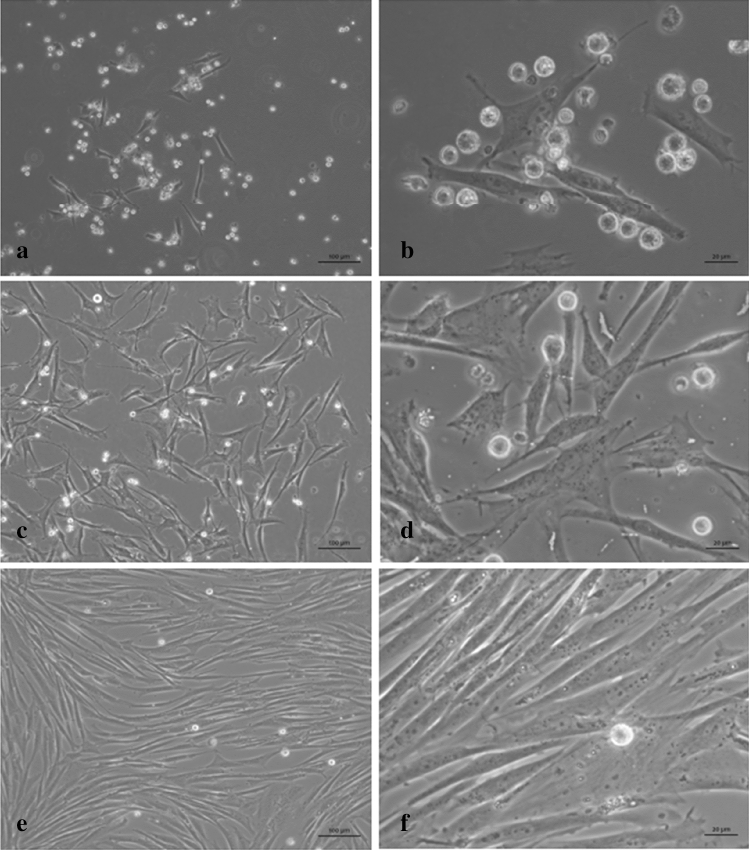
Microscopic features of canine MCT culture sample H334-16. (**a**,**b**) Early passage (P0) 4 days after initiation of culture, showing small clumps as well as individual neoplastic mast cells in the supernatant in association with the adherent layer. (**c**,**d**) 14 days of cultivation (P1 passage), with primary mast cells overlaying fibroblasts. (**e**,**f**) 34 days of cultivation, showing low number of viable mast cells in the supernatant and the fibroblasts tending to confluence. Phase contrast microscope. Bars = 100 µm (**a**,**c**,**e**) and 20 µm (**b**,**d**,**f**).

To observe the natural course of primary canine MCT cells in vitro, we harvested thirteen samples from eleven dogs (samples 1–13) immediately after the excisional biopsy, and monitored cell progression for up to 11 weeks by phase contrast microscope imaging analyses and cell viability measurement. Next, we demonstrated that all canine MCT primary cultures were composed by two major cell types: non-adherent cells (mast cells) and adherent cells (fibroblast-like) (Fig. 1). The vast majority of neoplastic mast cells ceased to proliferate and begun to die under in vitro conditions. Adherent cells after the initial seeding were identified as fibroblast-like with progressive proliferation. In all tumour samples, adherent cells rapidly multiplied to form a confluent monolayer under the neoplastic mast cells in 7–14 days (Fig. 1).

**Figure 2:**
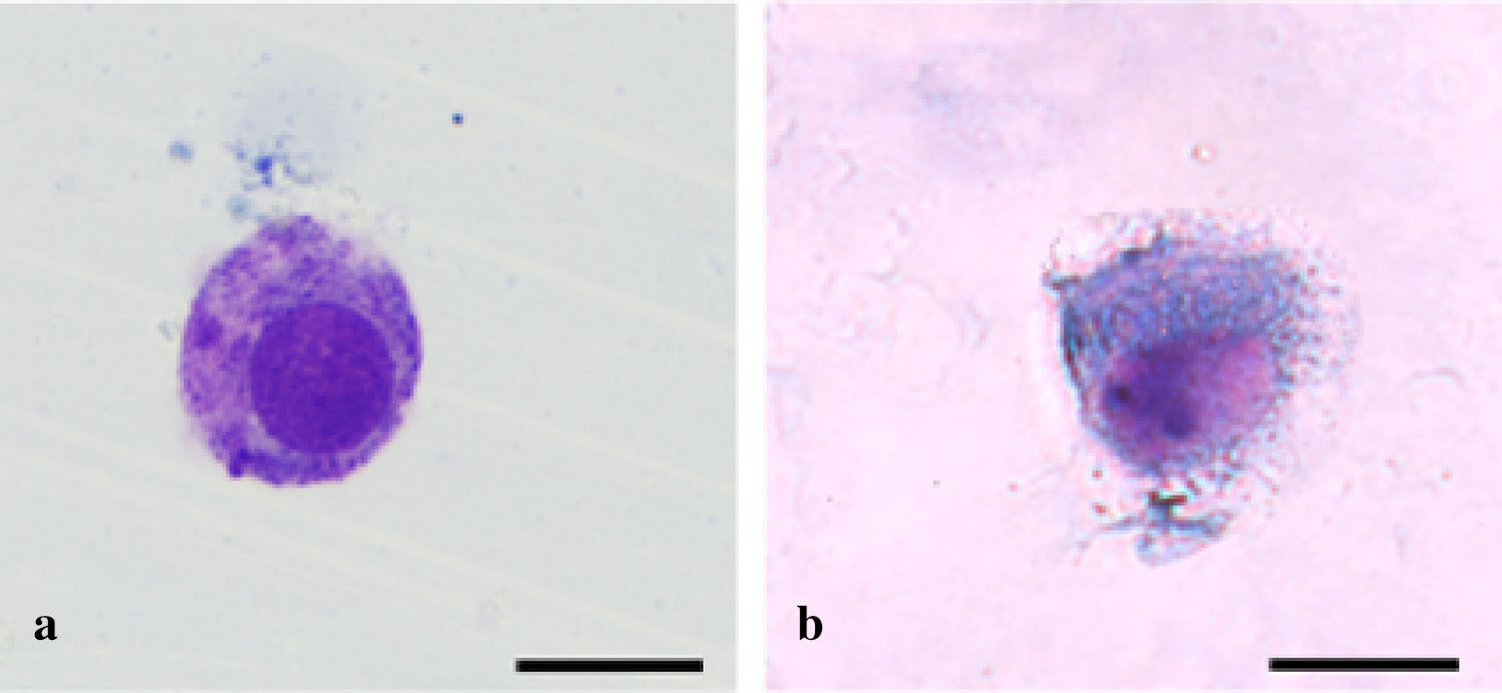
Mast cells 5 days after initiation of culture. (**a**) Romanowsky-stained preparation and (**b**) toluidine blue-stained preparation. Bars = 20 µm.

During all the passages of the primary culture, neoplastic mast cells were non-adherent cells isolated or forming floating aggregates. Mast cell population was maintained in clumps in continuity with the adherent layer with fibroblastic characteristics. At the beginning of the study and in each passage, neoplastic mast cells were characterized using Toluidine blue and Romanowsky stains to visualize the distinguished morphological features as the round-shaped cytoplasm, round to oval uniform-sized nuclei and variable quantities of metachromatic granules typical of cultured mast cells (Fig. 2). It was noted that these cells were progressively losing their granules in the culture over time.

### Phenotypic properties of adherent cells

**Figure 3:**
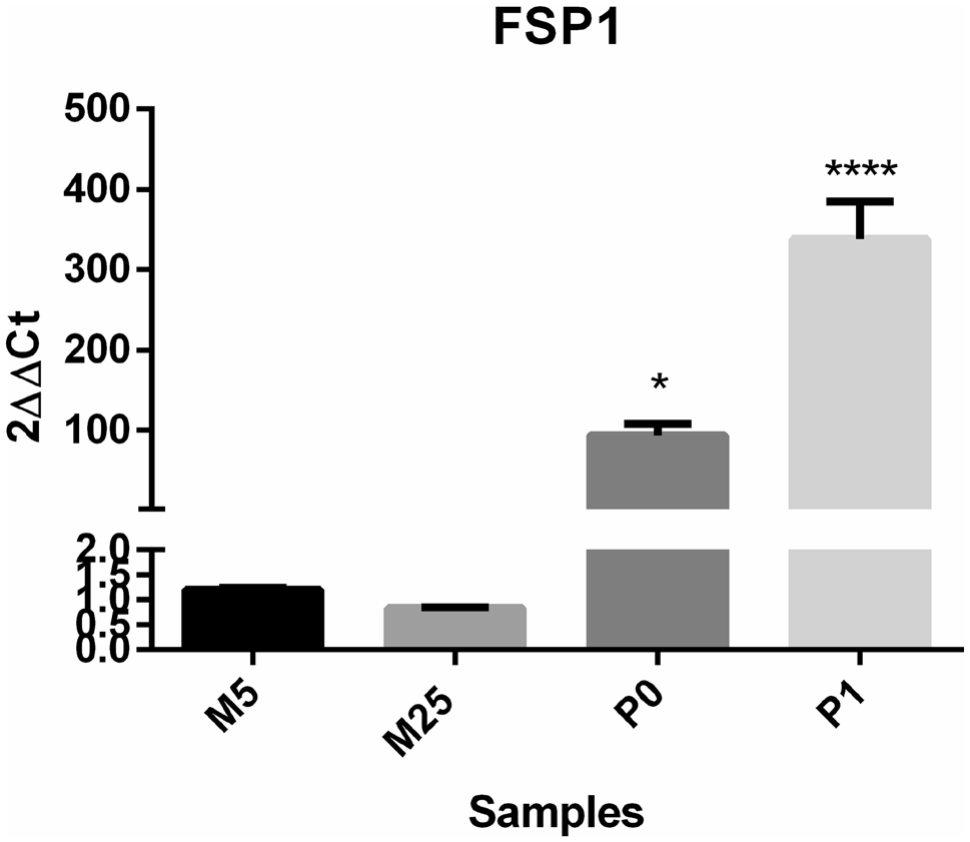
Fibroblast primary cultures exhibited significantly higher levels of FSP1 expression in comparison with canine mammary cancer cells (M5 and M25). M5 and M25 were used as reference samples to 2^ΔΔCt^ analysis. The 18S gene was used as the housekeeping gene (*p < 0.05; ****p < 0.0001; one way ANOVA followed by Tukey’s multiple comparison test).

We further analysed the phenotypes of the adherent cells differentiated into spindle-shape morphology. The success rate to generate fibroblastic-type cells in primary MCT cultures was 100%. Before distinguishing adherent cells in CAFs and non-cancer-associated fibroblasts (NCAFs), we characterized the fibroblast primary cultures by reverse transcription polymerase chain reaction (RT-PCR) using the Fibroblast-specific Protein 1 messenger RNA (FSP1 mRNA). Adherent cells from two tumour samples were evaluated on P0 and P1 and both displayed higher expression of this gene in comparison with canine cancer cells (M5 and M25) (Table 1; Fig. 3). We also characterized the other fibroblast primary cultures using RNA-seq data. Using global gene expression, we observed a strong cluster difference between fibroblasts and canine cancer cells (Supplementary Figs. S1 and S2). In addition, differential gene expression analysis showed that fibroblast-associated genes FSP1 and FAP are upregulated in fibroblast primary cultures in comparison with canine cancer cells lines (Table 2).

**Table 1:** FSP1 mRNA levels measured by real-time PCR and normalized by 18S ribosomal RNA (rRNA).

Sample	FSP1 Ct Mean	18S Ct Mean	2^ΔΔCt^ Mean
334–16 (P0)	32.61 ± 0.29	27.28 ± 0.08	93.92 ± 13.86
346–16 (P1)	23.77 ± 0.008	20.29 ± 0.19	338.46 ± 46.58
M5	23.06 ± 0.06	11.45 ± 0.02	1.20 ± 0.03
M25	23.24 ± 0.03	11.11 ± 0.007	0.83 ± 0.01

Ct (*cycle threshold*) values to FSP1 and 18S of P0, P1, M5 and M25 cells are observed as mean ± Sd.

**Table 2:** FSP1 and FAP mRNA levels measured by RNA-seq analysis.

Ensemble ID	Gene name	LogFC	FDR
ENSCAFG00000017550	FSP1 (S100A4)	− 3.3209	2.03E−31
ENSCAF00000010387	FAP	− 8.1810	5.71E−18

Comparisons were performed between canine cancer cells (M5 and M25) and adherent fibroblasts (H250, H346, H395, H401, H402 and H1171). Canine cancer cells were used as reference samples to logFold-Change (logFC) analysis. Both FSP1 and FAP genes are significantly upregulated in fibroblasts as shown by the logFC values.

**Figure 4:**
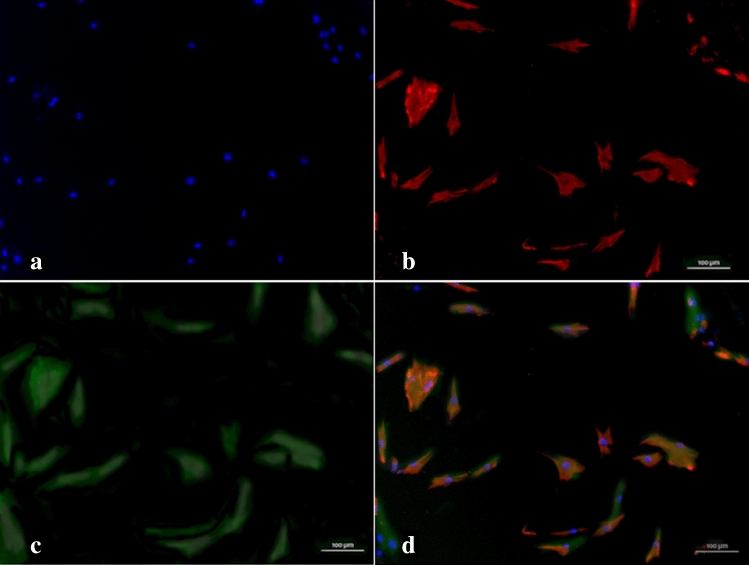
Representative immunofluorescence images of myofibroblasts from canine MCT culture. (**a**) Nuclei were stained with DAPI (4′,6-diamidino-2-phenylindole dihydrochloride; blue). (**b**) Adherent cells in MCT culture stained with vimentin antibody (red) and (**c**) anti-α smooth muscle actin (α-SMA) antibody (Phalloidin-FITC, green). (**d**) The merged image shows overlapping expression (original magnification ×400).

Immunofluorescent staining of adherent cells with fibroblasts phenotype (a spindle, flattened-spindle, or less frequently triangular shape, with frequent long terminal threadlike processes) revealed myofibroblast features, including vimentin and α-SMA expression (Fig. 4).

### Cell viability in primary cocultures

**Figure 5:**
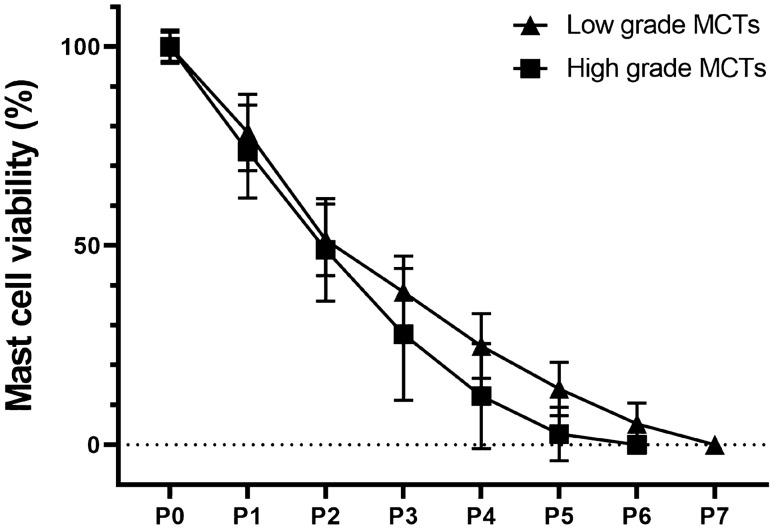
MCTs classified as low- and high-grade cultures at different passages along 11 weeks. Neoplastic mast cell culture (conditioned by fibroblasts) viability *versus* passages—percentage of viable mast cells during the passages of thirteen independent cultures from individual lesions. Samples are specified in Supplementary Table S1. Viability was determined by the Trypan blue exclusion method. Mean and Standard Deviation are represented in the graph, and no difference between low- and high-grade cultures were observed in any passage.

We evaluated cell viability of each MCT to determine the maintenance time of viable neoplastic mast cells in vitro cocultured with CAFs. Thus, non-adherent cells were stained with Trypan blue exclusion test to quantify the viable mast cells in the supernatant in each passage. Cultures were seeded with a total number of cells ranged from 5.5 × 10^5^ to 3.6 × 10^6^ cells/mL (mean 1.5 × 10^6^ ± 1,034,832). Neoplastic mast cells remained viable for a period that ranged from 30 to 75 days (average time of 57.7 days). In all samples, viable mast cells decreased during culture passage in number and percentage of cells (Fig. 5) which was consistent with the observed in the analysis of the different passages in the microscope. The number and percentage of viable mast cells obtained after digestion of skin MCT for each lesion and passage can be seen in Supplementary Tables S2 to S14 (Supplementary Materials).

**Figure 6:**
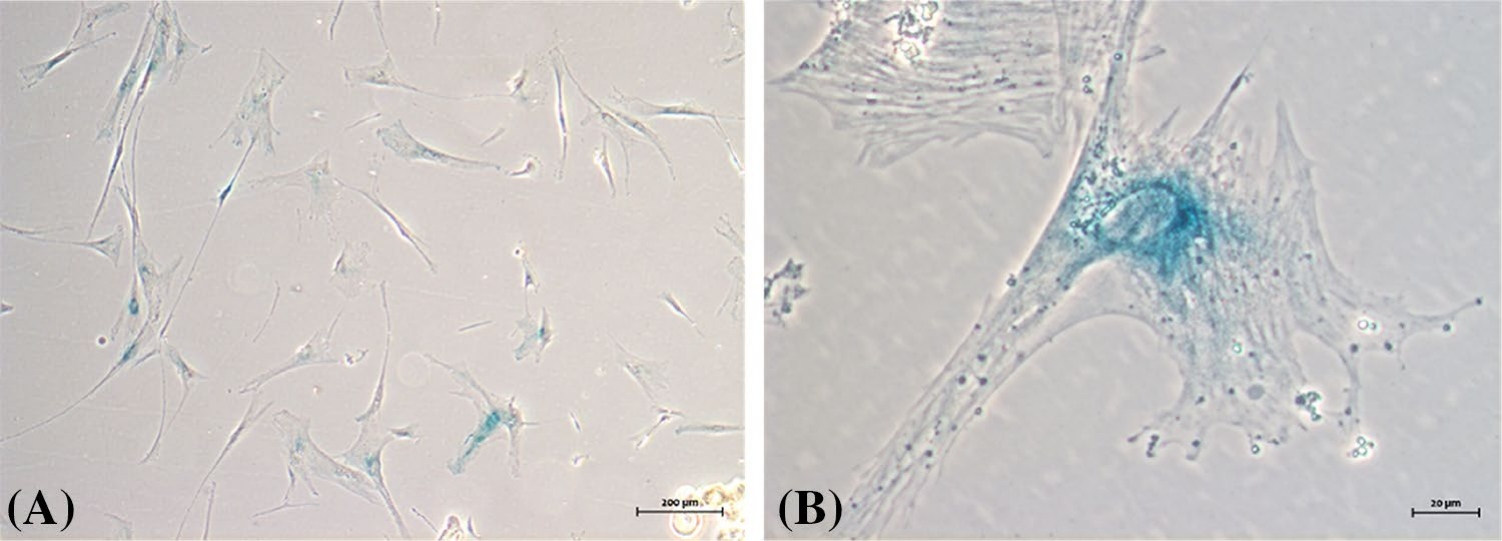
Cytochemical detection of Senescence-Associated β-Galactosidase (SA-β-Gal) activity in stromal fibroblasts of canine MCTs (Sample H05-16). Cells maintained in culture for 2 months (P5). (**A**) Positivity in most cells through the formation of blue precipitate in the cytoplasm. Bright field microscopy. (**B**) Fibroblasts demonstrating the most intense staining perinuclear. Phase contrast microscopy.

In addition to cell viability, adherent cells were examined using beta-galactosidase (SA-β-Gal) activity assay. All samples in middle and late passage cultures (starting from P4), CAFs and NCAFs ceased to be confluent and begun to change their phenotype to an enlarged cell morphology with depleted replicative potential. Senescence-associated beta-galactosidase (SA-β-Gal) activity was measured in late passages, with several strongly positive cells for SA-β-Gal, mainly in perinuclear area (Fig. 6). Notably, passages showing a high number of SA-β-Gal-positive adherent cells presented loss of replicative capacity, revealed by failure to reach confluence.

### Cocultures and transwell assay

Dissociated cells from canine MCTs, seeded and cultured for up to 48 h could be distinguished between two cell subpopulations: adherent (CAFs and NCAFs) and non-adherent (mast cells). To address the importance of stromal cells for neoplastic mast cells in vitro maintenance, we performed transwell experiments to observe physical and/or chemical effects of CAFs and NCAFs on the canine tumoral mast cells viability. Four co-culture conditions were tested: tumour mast cells and fibroblast direct cell-to-cell contact; tumour mast cells and fibroblast physically separated by the insert; tumour mast cells in monoculture supplemented with complete conditioned medium (CCM); and tumour mast cells in monoculture supplemented with basic medium consisting only of FBS and antibiotics (CDMEM-F12). Four dogs with single MCTs (two low-grade and two high-grade) were selected in these experiments to ensure we were dealing with primary tumours.

**Figure 7:**
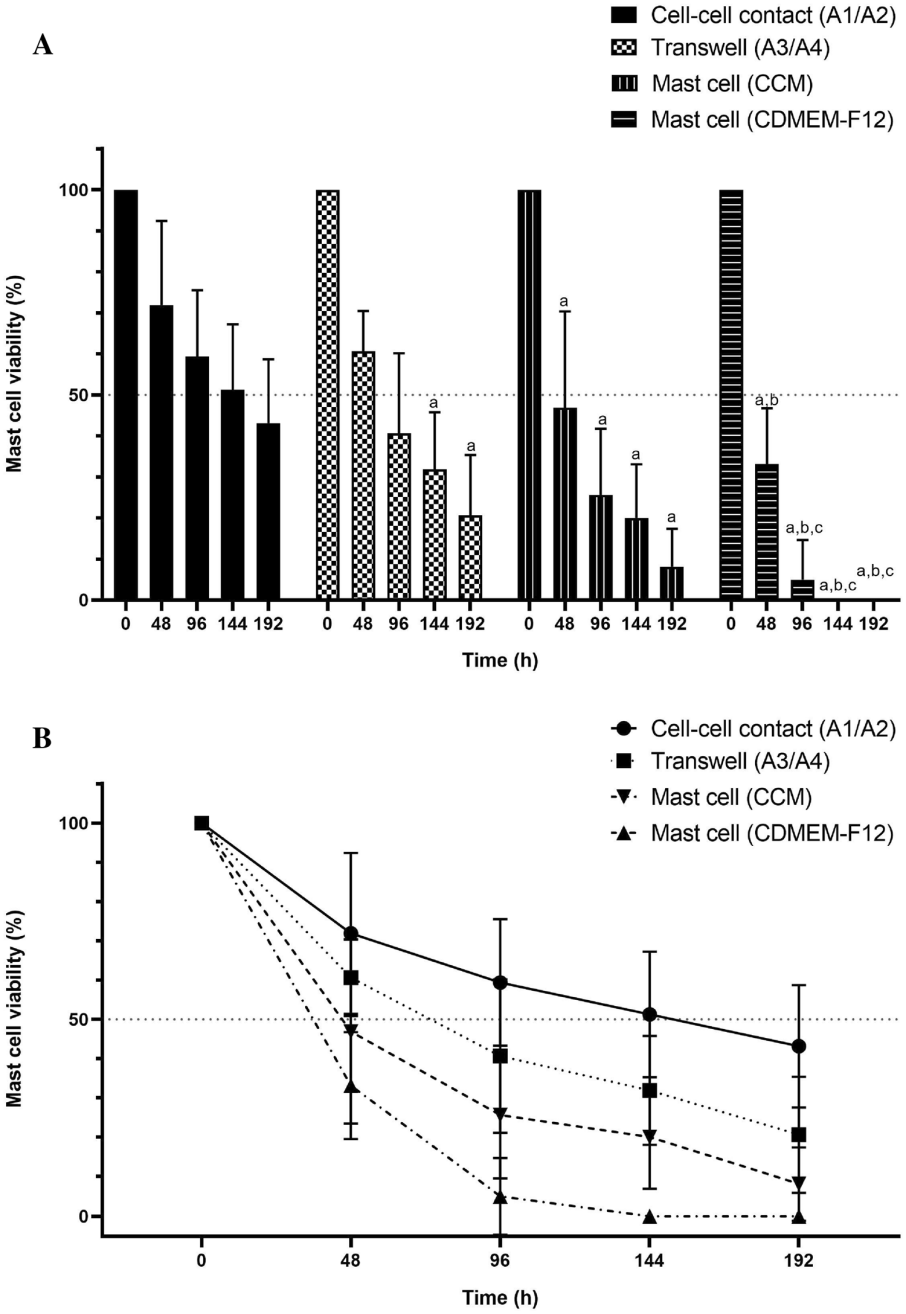
Results of the co-culture assay in four MCT samples (H550-17, H401-18, H1171-18 and H1249-18). (**A**) Each bar represents the mean and standard deviation of duplicate measurements. Canine mast cells cultured in cell-to-cell contact with an adherent layer of stromal fibroblasts showed the highest average number of viable cells in each time-point (a = *P* < 0.05 compared with cell–cell contact condition; b = *P* < 0.05 compared with Transwell co-culture system; c = *P* < 0.05 compared with mast cell in monoculture with CCM). (**B**) Tumour mast cell viability in each condition along 192 h of cultivation.

Thus, by using neoplastic adherent and mast cells from the same tumour, we observed that both time and culture condition contributed to variation in survival of co-cultured cells (*P* < 0.01). At all time-points, viability of neoplastic mast cells was significantly higher in the cell-to-cell contact condition compared to mast cell monocultures in both CDMEM-F12 and CCM, while differences between cell-to-cell and transwell conditions were only observed from 144 h onwards (Fig. 7A). In monocultures, the use of CCM led to a significantly longer mast cell viability when compared to mast cells supplemented with CDMEM-F12, while no significant changes were observed between CCM and transwell conditions at any time-point. Finally, mast cells in monoculture without any physical or chemical influence of adhered fibroblasts presented the lowest viability of all conditions, in which approximately 90% of cells died after 96 h of cultivation (Fig. 7B).

**Figure 8:**
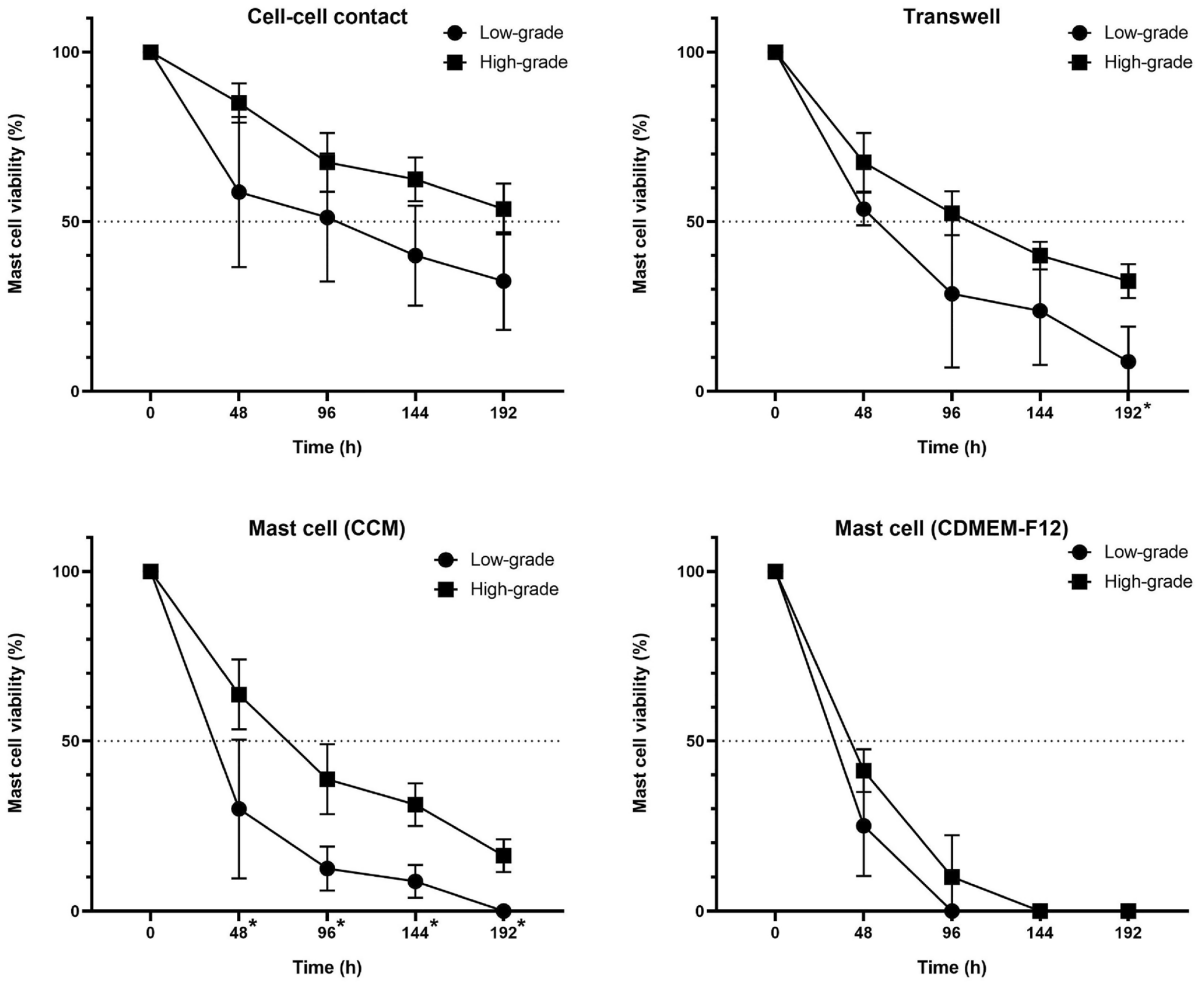
Comparison of neoplastic mast cells survival from low- and high-grade MCTs in each co-culture condition. Graphs show median ± SD of mast cells viability up to 192 h. Statistical significance (*) was observed only in the transwell assay after 192 h (q value = 0.019) and in cultures supplied with complete cultured medium (CCM) after 48 (q value = 0.0268), 96 (q value = 0.007), 144 (q value = 0.0026) and 192 h (q value = 0.002).

To investigate the possibility of neoplastic mast cells in vitro behaviour to be depended of histopathological grade, we compared low-grade and high-grade tumours in all four culture conditions. Differences between the two groups were found in all selected time-points in mast cells monoculture supplemented with CCM, where cells originated from low-grade samples presented a lower viability than those isolated from high-grade tumours (Fig. 8). The same pattern was found in the transwell system assay, although the significant difference was observed only after 192 h of cultivation. No differences were found in cell-to-cell contact and CDMEM-F12 monoculture conditions.

## Discussion

In the present study, we demonstrated the inability of canine neoplastic mast cells to remain viable for long periods in vitro without the addition of growth factors or in vivo passages in mice, corroborating the observations of Pinello et al.^31^. The mast cells adhered to the fibroblasts but did not grow. The main reports on the establishment of cell lines from canine MCTs in continuous culture and with no previous passage in mice are: HRMC^32^, CM-MC, VI-MC, CoMS^33,34^, MPT-1^35^, MPT-1.2 cells^36^ and MPT-2^37^. However, in these studies, important characteristics were not described in detail, such as the number of passages with viable cells, the culture purification methods, culture conditions, and presence of fibroblasts or other cells in coculture. So far, long-term cultures of canine cutaneous mast cells are only possible with combined in vitro and in vivo approaches^8–12^.

The notion that the mast cell phenotype can be importantly regulated by the microenvironment has been the subject of much discussion and interest. There are many possible factors that might have an effect on mast cell phenotype and influence mast cell in vitro survival^37–39^, but CAFs are one of the most important components of the tumour microenvironment^41^. This feature has also been observed in human and murine mast cell lines^42,43^ and can be induced by cell-to-cell aggregation and mechanical handling effects^5^. In this study, neoplastic mast cells grew in suspension as single cells or in clusters, and this condition was similar to that observed in other canine MCT cultures^33,35,45^.

In vivo, fibroblasts from cancer stroma undergo a myofibroblast transformation as part of the cellular response to neoplastic growth^45^. MCT-derived CAFs are fundamental regulators of in vitro tumour maintenance and recognized essentially, but not exclusively, based on α-SMA expression^46,47^. We confirmed the presence of myofibroblasts in canine MCT cultures in agreement with our previous findings in tumour tissue^29^ and the observations of Giuliano et al.^28^, which had identified CAFs in histopathological samples of canine MCTs. Fibroblast-secreted protein-1 (FSP1) produced by CAFs is another important factor in promoting the cancer cell growth that may have helped mast cell in vitro viability.

Knowing that neoplastic mast cell survival in vitro depends, in part, on the interaction with adhered cells, we employed different culture systems to elucidate the type of signalling. The mechanism of interaction across the cytoplasmic membrane should be considered, since mast cells form robust adhesions with fibroblasts in the coculture conditions^48^ that are not mediated by known integrin or cadherin receptors^49^. Notably, mast cells in monoculture normally grow in suspension and do not strongly adhere to plastic culture surfaces^50^. Therefore, we considered that mast cell attachment to fibroblasts with intercellular signals may be important for in vitro mast cell short-term maintenance. We also believe that this cellular communication occurs through heterogeneous gap junction channels between neoplastic mast cells and CAFs^51–54^ promoting enhanced interleukin release^48^.

Upon activation, CAFs secrete a vast repertoire of growth factors, such as hepatocyte (HGF), epidermal (EGF), insulin-like (IGF) growth factors and cytokines, including CXCL12 and IL-6^55^. It was reported that IL-6 expression by CAFs is up to 100-fold higher than normal fibroblasts^56^. Highlighting the importance of physical interaction between cells, fibroblasts are capable of producing IL-6 when stimulated directly by mast cell via cell-to-cell interaction in coculture^57–59^, and less efficiently by mast cell mediators^60^. IL-6 derived from CAFs and NCAFs is responsible for suppressing apoptosis in human intestinal mast cells^60^ and hepatic mast cells^61^ under cell-to-cell contact condition. When bone marrow derived mast cells were cultured with a fibroblast monolayer, the IL-6 family of cytokines (Il-6, IL-11, OSM and LIF) induced proliferation of mast cells, but only when the fibroblasts were present. Thus, given that neoplastic mast cells and adherent cells were physically separated, it is likely that paracrine signalling through the pores of the membrane may be influencing mast cell population.

We also found that monocultures of canine neoplastic mast cells are not viable for more than four days in the absence of fibroblasts or their soluble factors. Similar results were obtained in vitro for mast cells cultured without a stromal adherent cell layer^16,31,39,43,62^. Interestingly, we did not detect differences between the two culture conditions with mast cells receiving conditioned medium (CCM), i.e., transwell assay and mast cells in monoculture with conditioned medium obtained from primary MCTs cultures supernatants. However, both conditions supported a superior number of viable mast cells compared to CDMEM-F12. We could attribute this finding to paracrine signalling between fibroblasts and mast cells.

The main limitations of the present study were the lack of knowledge about the exact composition of the conditioned medium and effective molecules secreted by the stromal fibroblasts in coculture. We believe that neoplastic mast cell survival in vitro depends, in part, on the interaction with the adhered fibroblasts and CAFs. Fibroblasts are known to secrete Stem Cell Factor (SCF), which is the main growth factor for mast cells^38,40^ and an essential factor for mast cell growth in rodent and human mast cells cultures^38,62–64^. In addition, several studies with mast cell cultures indicated the requirement of both IL-3 and SCF to expansion and maturation, even in the presence of stromal cells^62,65^. Literature data suggest that the establishment of mast cell lines in permanent culture in vitro with no passages by nude mice should depend on SCF supplemented medium^40,64^ and the presence of stromal cells may assist in the maintenance of mast cells in vitro^14,17,62^. Finally, we need to consider that the partial decrease of mast cells in passages may in part reflect cell loss during the harvesting procedure^5^ or a relatively low density of the cultures.

The skin tumours used in this study provided a fibroblast population that declines in vitro with serial passaging, with increasing number of cells entering replicative senescence^66–68^. We believe that the earlier onset of cell culture senescence may be due to the age of the cell culture donors^67,69,70^. This process is thought to be caused by aging and these “aged” skin fibroblasts with limited replicative potential may be due to canine cellular aging in vivo, since the mean age of the dogs included in the present study was around 7.5 years.

We have also shown the presence of myofibroblasts in canine MCT cultures. This finding is in concordance with the observations of Giuliano et al.^28^, which demonstrated that fibroblasts from canine MCT stroma have CAF phenotype and was correlated positively with high grade, high mitotic index and high Ki67 expression. We believe that this feature might be important to the understanding of the different clinical responses to chemotherapy, since CAFs could influence the drug-sensitivity of cancer cells^71^. Here, we have also shown that histopathological grade, which is related to the degree of cell differentiation, might have influenced survival in monocultures supplemented with CCM: mast cell viability was slightly higher in high-grade MCT-derived cultures. Although preliminary, due to the low number of samples, these results are interesting in the light of the different experimental approaches in literature, and could help to explain, for example, the higher invasion and metastatic rates in these cases, since neoplastic mast cells could be less dependent on cell-to-cell contact with fibroblasts. To the best of our knowledge, no previously published work compares the in vitro behaviour of tumours with different histopathological grades. Variability in mast cell phenotype as, for example, differences in the content of cellular proteases, reflects the action of the local microenvironment^40^. The mechanisms underlying the relationship between the grade of malignancy and the culture conditions remain unclear and deserve future investigation.

## Conclusion

In conclusion, reproducible development of canine tumour-derived mast cells by a long-term culture may need in vivo passages, fibroblasts interactions and/or supplemented medium with growth factors. Cultures without artificial stimuli would be a major breakthrough to understand canine MCT biology. Here, we showed that stromal MCT fibroblasts present a CAF phenotype in vitro when cocultured with neoplastic mast cells, constituting a promising and versatile in vitro model to study various aspects of MCTs. Fibroblasts and CAFs transcription factors could be important for MCT malignant phenotypes, through direct or indirect stimuli, in which different stroma cells cultured together communicate to create a tumour-permissive microenvironment and thus may contribute to the maintenance of cancer mast cells. Signalling mechanisms involved in these interactions are attractive therapeutic targets to block canine MCT progression. Ongoing studies in our laboratory aim to investigate canine mast cells and tumoral stroma cells interactions.

## Materials and methods

### Tumour samples

Mast cell tumour samples were obtained from dogs that underwent surgery at the Veterinary Hospitals of the Faculty of Animal Science and Food Engineering of the University of São Paulo (FZEA-USP) and the Octávio Bastos Foundation University (UNIFEOB), and private veterinary clinics, which agreed to participate in the present study. All surgical procedures were performed as part of the treatment, aiming the cure. Inclusion criteria were confirmed histopathological diagnosis of cutaneous MCT, no previous antineoplastic treatment (radio or chemotherapy) and availability of complete medical records. All experiments were approved by and performed in accordance with the guidelines and regulations of the Ethic Committee on Animal Use of the Faculty of Veterinary Medicine and Animal Science (protocol #CEUA/5637040718). The reporting in the manuscript follows the recommendations in the ARRIVE guidelines. Informed written consent was obtained from the owner of each dog whose MCT biopsy was included in this study. Patient treatment was unaffected by the study. All samples had a representative fragment placed in 10% neutral buffered formalin, processed routinely and embedded in paraffin. Four-µm sections were stained with haematoxylin and eosin and MCTs were graded according to Kiupel et al. ^30^.

### Dissociation and primary cell co-culture

Canine MCT samples obtained immediately after excisional biopsy under sterile conditions were placed in sterile transport medium at 4 °C consisting of Dulbecco's Modified Eagle Medium/Nutrient Mixture F-12 (DMEM-F12, Thermo Fisher, Fremont, California, USA), antibiotics penicillin G/streptomycin (200 U/mL, Sigma-Aldrich and 100 U/mL; Sigma-Aldrich, respectively) and 15% fetal bovine serum (FBS) until arrival at the laboratory. Subcutaneous fat tissue was removed and the tumours were minced finely into approximately 0.5–1.0 mm^3^ fragments and incubated in DMEM-F12 with an enzyme mixture: 200 U/mL collagenase type I (Sigma-Aldrich, St. Louis, MO, USA) and 100 U/mL hyaluronidase (Sigma-Aldrich, St. Louis, MO, USA) supplemented with 15% FBS and 2% antibiotics penicillin/streptomycin. After 180 min in water bath at 37 °C, the disaggregated tumour was filtered through a 100-μm filter followed by filtration through a 40-μm mesh (Cell Strainer, BD Biosciences). Cells dispersed by this procedure were centrifuged at 1500 rpm for 5 min at 25 °C and the sediment was re-suspended in DMEM-F12 supplemented with 15% of FBS and 1% of penicillin/streptomycin, denominated in this study as CDMEM-F12 for experimental purposes.

The cells obtained from the MCT were maintained at 37 °C in a 5% CO_2_ atmosphere in two 75 mm^2^ tissue culture flasks/sample with 10 mL of complete medium DMEM-F12. The medium was renewed every 5–7 days, when adherent cells reached confluence of approximately 70%. The supernatant with nonadherent cells was removed, centrifuged at 1500 rpm for 5 min, resuspended in complete DMEM-F12 medium and then passed to a sterile culture bottle flask with adherent cells at a density of 10^5^ cells per mL. The coculture was evaluated daily by Eclipse-TS100 microscope equipped with a Nikon camera (Nikon, Tokyo, Japan).

At each passage, a supernatant aliquot of 100 μL was harvested, centrifuged at 1500 rpm for 5 min and the pellet placed on a glass slide. Mast cells were identified by 0.05% toluidine blue (Sigma-Aldrich Corp.) stain and with Romanowsky stain (Diff*-*Quik solution*,* Dade Behring Inc*.,* Düdingen, Switzerland). Cell number was assessed in a Neubauer haemocytometer and cell viability was checked with Trypan Blue Exclusion Test according to Strober^72^, using an optical microscope (Eclipse TS100, Nikon, Japan).

### Quantitative real-time polymerase chain reaction analysis for Fibroblast-specific Protein 1 (FSP 1)

Total RNA was extracted from adherent cells derived from cultured canine cutaneous MCT at the initial (P0) and first passage (P1) using TRIzol reagent (Thermo) according to the manufacturer's instructions. NanoDrop2000 (Thermo Scientific, USA) and RNA integrity number (RIN) were used to verify the amount and integrity of the extracted RNA following manufacturer's protocol (RNA 6000 Nano kit, 2100 Bioanalyzer, Agilent Technologies, USA). Samples with A260/A280 ratio between 1.8 and 2.1 and RIN superior than 8 were considered appropriate for use. Subsequently, reverse transcription into cDNA was made using the commercial kit “High-Capacity cDNA Reverse Transcription Kit” (Thermo) at 37 °C for 120 min and 75 °C for 5 min in a PCR thermal cycler. Specific primers for Fibroblast-specific Protein 1 (FSP1) gene were designed with Primer-BLAST^73^.

Real-time quantitative polymerase chain reaction (qRT-PCR) was performed with Fast SYBR Green Master Mix in a final volume of 10 μL in the presence of primers for canine FSP1 gene: 5′-TCCTCATCTCTTCTCCTTCTTGGT-3′ (forward) and 5′-TGAACTTGTCACCCTCCTTGC-3′ (reverse) in a final concentration of 50 nM. Primers were design with Primer-Blast^73^ and the possibility of dimers and hairpins were verified using AutoDimer software^74^. Primers were also analysed by in silico PCR to confirm specificity (https://genome.ucsc.edu/cgi-bin/hgPcr). Gene expression analysis were performed by qRT-PCR using a StepOne System (Thermo Fisher Scientific). Conditions for qPCR were as follows: 95 °C for 20 s; 40 cycles at 95 °C for 3 s for denaturation, 60 °C for 30 s for anneal/extend; melt curve analysis was performed at 95 °C for 15 s and 60 °C for 60 s. Relative expression levels of the target gene were normalized to the housekeeping gene (18S ribosomal RNA) and determined using the 2∆∆Ct method^75^. The experiment was performed twice and in biological triplicates.

### RNA-seq and differential expression

RNA libraries were constructed using the TruSeq Stranded mRNA LT Sample Prep Protocol and sequenced on Illumina HiSeq. 2500 equipment in a HiSeq Flow Cell v4 using HiSeq SBS Kit v4 (2 × 100 pb). Sequencing quality was evaluated using the software FastQC (http://www.bioinformatics.babraham.ac.uk/projects/fastqc) and no additional filter was performed. Sequence alignment against the canine reference genome (CanFam3.1) was performed using STAR^76^, according to the standard parameters and including the annotation file (Ensembl release 89). Secondary alignments, duplicated reads and reads failing vendor quality checks were removed using Samtools^77^. Alignment quality was confirmed using Qualimap^78^. Gene expression was estimated by read counts using HTseq^79^ and normalized as counts per million reads (CPM). Only genes presenting at least 1 CPM were kept for differential expression (DE) analysis. Groups were divided between canine cancer cells (M5 and M25) and Fibroblasts (H250, H346, H395, H401, H402, and H1171). DE between the groups was performed using EdgeR package^80^ on R environment, based on negative binomial distribution. Benjamini–Hochberg procedure was used to control the false discovery rate (FDR) and transcripts presenting FDR ≤ 0.01 and log-Fold Change (logFC) > 1 or < − 1 were considered differential expressed (DE).

### In vitro characterization of adherent cells: immunofluorescence for α-smooth muscle actin (α-SMA) and vimentin

Fibroblasts derived from the MCT stroma were grown on glass coverslips right after tumour dissociation (P0) in complete DMEM F12 medium. Adherent cells were washed three times with PBS and then fixed in 4% paraformaldehyde solution (in PBS) for 10 min at room temperature. After washing with PBS, unspecific antigens were blocked for 45 min with a 5% fat-free skim milk solution at room temperature. Coverslips containing fixed cells were first incubated at 4 °C in in humid chamber with the primary anti-vimentin antibody conjugated to fluorophore Alexa Fluor 647 (clone V9, cod. ab195878, Abcam, USA) diluted 1:100, for 16 h (overnight), washed and incubated for 60 min in a 1:500 dilution of monoclonal anti-alpha smooth muscle actin antibody conjugated to fluorescein isocyanate (FITC) (clone 1A4, Sigma-Aldrich, St Louis, MO, USA) at room temperature. After rinsing in PBS, nuclei were stained with DAPI. For the negative control, the antibodies were replaced with PBS. Coverslips were mounted on glass slides using ProLong solution (Thermo Scientific, USA) and observed immediately with an inverted fluorescence microscope (ZEISS—Axio Vert.A1) and photographed with a coupled camera AxioCam 503 attached using 540 nm wavelength filter for the observation of smooth muscle actin filaments (FITC) and 670–695 wavelength filter for visualization of vimentin (Alexa Fluor 647) and 358 nm for observation of the DAPI-labelled nuclei. Photomicrographs were taken using a ZEISS ZEN 2 Microscope Software (Carl Zeiss, Jena, Germany). All double immunofluorescence results were verified by single labelling techniques using direct (vimentin and alpha-actinin) fluorescence.

### Adherent cells senescence assay

To assess cellular senescence in cultured fibroblast-like cells presenting a rapid decreasing in growth rate, we measured endogenous β-galactosidase activity as previously reported^81^. Fibroblasts used in this experiment were between passages 5 and 7. Briefly, cells were washed in PBS, fixed in 4% paraformaldehyde solution for 5 min at room temperature and incubated overnight at 37 °C with X-gal chromogenic substrate at pH 6.0 following the conventional protocol for SA-β-gal staining. The proportion of cells positive for β-gal activity can be easily determined by counting the number of blue cells in the total population. The cytochemical staining in senescent fibroblasts-like cells were observed by inverted ZEISS Axio Vert.A1 microscope and photographed with a coupled camera Axio Cam 503.

### Co-culture transwell assay

Initially, cell populations obtained from primary tumours were cultivated in contact for 5–7 days. For each lesion, we obtained two bottles of primary culture at P0: one provided the neoplastic mast cells (supernatant) and CAFs (adhered); the other was maintained for supplying the tumour tissue culture medium (TTCM). Tumour cells cultured in P1 were used to perform the coculture transwell assay. Mast cells were obtained from the supernatant of primary cultures and CAFs adhered to the bottom were detached with enzymatic dissociation for 4–6 min at 37 °C in Trypsin (TrypLE Express 1X, with phenol red, Thermo Fisher Scientific). Two different media were used: complete DMEM-F12 medium (CDMEM-F12) when both cell types were present in the same well and complete conditioned medium (CCM) in monoculture wells, that was composed by one part of complete DMEM-F12 10× concentrated and nine parts of TTCM.

All assays were performed in duplicate, using a 12-well plate/tumour. Some wells received cell culture inserts with translucent polyester membrane (Thincert, Greiner Bio-One, Kremsmünster, Austria). Pore sizes and density of the membrane were 0.4 µm and 2 × 10^6^ pores/cm^2^, respectively. In each well, 1 × l0^5^ neoplastic mast cells were seeded. CAFs were seeded at an initial density of 1 × l0^4^ cells/mL in a total medium volume of 1 mL/well.

**Figure 9:**
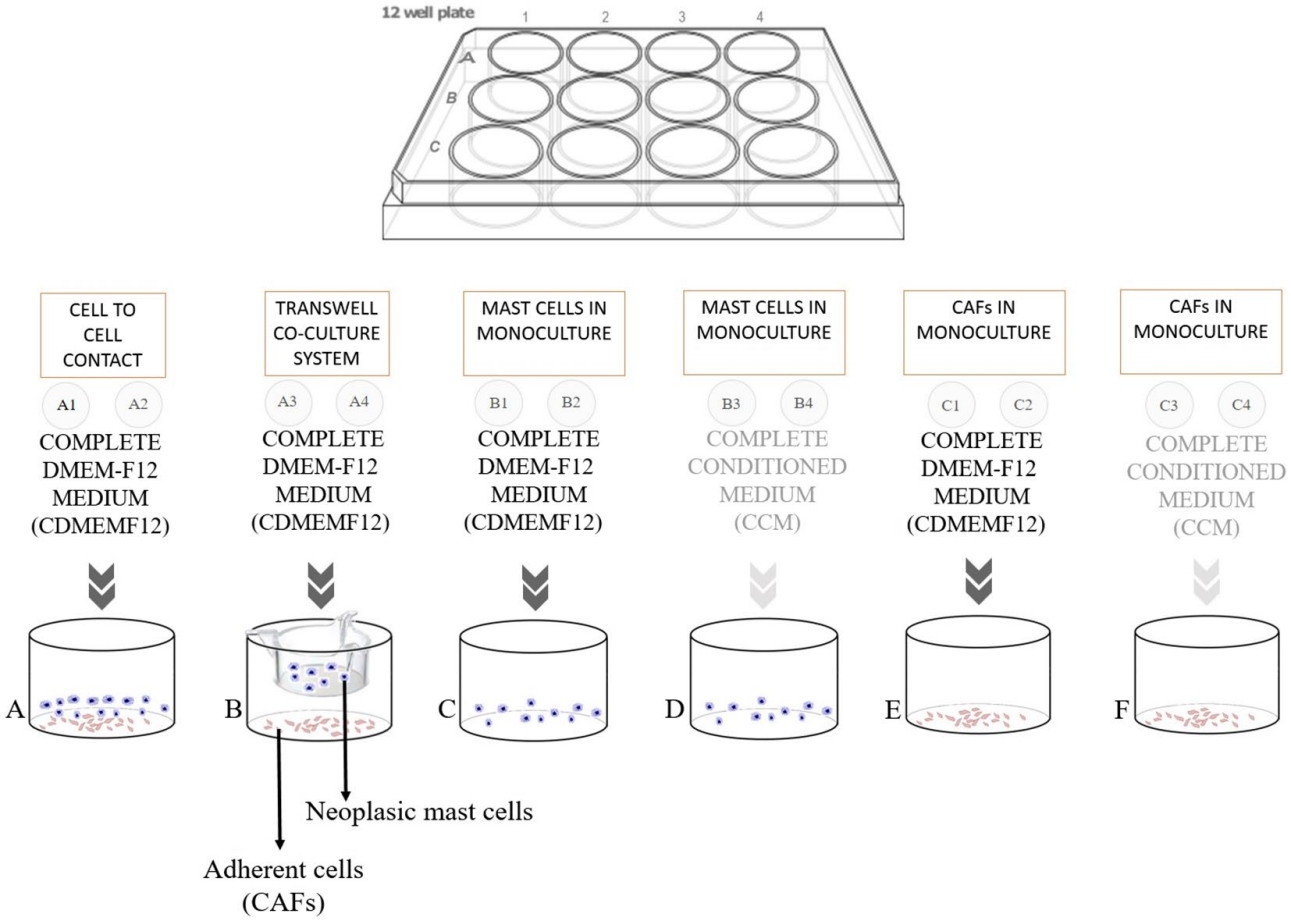
Coculture schematic model for the evaluation of neoplastic mast cells and stromal fibroblasts interactions. (**A**) Cell-to-cell coculture condition that allows the chemical and physical interaction between neoplastic mast cells and fibroblasts. (**B**) Transwell coculture system, with neoplastic mast cells and stromal fibroblasts in a condition that allows only chemical communication. Mast cells were added into the insert and fibroblasts into the well for adherence to the surface. (**C**) Mast cells in monoculture with complete DMEM-F12 medium and (**D**) with complete conditioned medium. (**E**) Fibroblasts in monoculture with complete DMEM-F12 medium and (**F**) with complete conditioned medium.

The experiments were conducted under the following conditions: (1) Cell-to-cell contact, with mast cells seeded in direct contact with fibroblasts in a complete DMEM-F12 medium (CDMEM-F12) (Fig. 9A); (2) Transwell coculture system using cell culture inserts (ThinCert, Greiner Bio-One), in which two cell populations were placed in different compartments, i.e., neoplastic mast cells in the upper compartment (insert) and fibroblasts in the lower compartment (adhered to plate surface), in CDMEM-F12 medium (Fig. 9B); (3) Mast cells in monoculture, with neoplastic mast cells cultured alone in suspension receiving CDMEM-F12 medium (Fig. 9C) or CCM (Fig. 9D); and (4) Fibroblasts in monoculture maintained with CDMEM-F12 medium (Fig. 9E) or with CCM (Fig. 9F). Cocultures were maintained for 8 days at 37 °C and 5% CO_2_, with daily observation using an inverted microscope (ZEISS Axio Vert.A1, Germany). Mast cell viability was determined by the Trypan blue exclusion method every 48 h with the removal of 10 μL supernatant of each insert/well with previous agitation. Cell counts and viability were examined at days 2, 4, 6 and 8. Adhered cells were not quantitatively evaluated (wells: C1, C2, C3 and C4). The experiment was maintained until death of all mast cells from some of the culture conditions.

### Statistical analysis

The number of non-adherent mast cells obtained in the primary co-culture assay were presented as the mean ± standard deviation (SD) for each sample. Normality was tested using the Kolmogorov–Smirnov method. Comparison of mast cell viability for high- and low-grade cultures conditioned by fibroblasts and between co-culture conditions was performed using multiple t-tests, corrected for multiple comparisons using the Holm–Sidak method (alpha = 0.05). Comparison of mast cells viability in the co-culture assay considering each condition were performed with a 2-way ANOVA test, followed by correction for multiple comparisons using Tukey’s statistical hypothesis testing. Significance level was defined as 0.05 (95% confidence interval). All statistical analyses were conducted using GraphPad Prism (GraphPad Software, CA, USA).

## Supplementary Information

Supplementary Information.

## Data Availability

The datasets generated during and/or analyzed during the current study are available from the corresponding author on reasonable request.
