# Supplementary material for: Intercellular interactions between mast cells and stromal fibroblasts obtained from canine cutaneous mast cell tumours
Source: Sci Rep. 2021 Dec 13;11:23881. doi: 10.1038/s41598-021-03390-w (PMC8668961; doi:10.1038/s41598-021-03390-w)
Supplement: Supplementary file 1 — Supplementary Information. [file 41598_2021_3390_MOESM1_ESM.pdf]

SUPPLEMENTARY MATERIALS

TABLES

Table S1. Summary of Clinical and Histopathological data of 14 tumours obtained from 12 dogs with MCTs submitted to the coculture and transwell analyses. Breed, age, gender, location of the lesions and histopathological grades, with their respective identification number. Sample 401-18 was not used for the assessment of cell viability along passages.

SAMPLE ID	BREED	AGE (years)	GENDER	TUMOR LOCATION	GRADE [27]
5/16[a]	mixed breed	8	male	trunk	Low-grade
6/16[a]	mixed breed	8	male	limbs and tail	Low-grade
346-16	Labrador	5	female	trunk	Low-grade
19-18	Labrador	6	male	limbs and tail	Low-grade
1171-18[b]*	mixed breed	9	female	trunk	Low-grade
1172-18[b]	mixed breed	9	female	trunk	Low-grade
1249-18*	Australian Cattle Dog	3	female	head	Low-grade
232-16	mixed breed	4	female	abdomen	High-grade
334-16	mixed breed	10	male	inguinal	High-grade
395-17	mixed breed	7	male	limbs and tail	High-grade
550-17*	Boxer	10	female	abdomen	High-grade
603-17	Brazilian Terrier	14	female	limbs and tail	High-grade
816-18	Boxer	6	female	limbs and tail	High-grade
401-18*	Brazilian Terrier	9	male	limb	High-grade

ID = identification number in the pathology laboratory.
[a,b] lesions from the same patient
* tumour which primary cells were used for co-culture experiments

Table S2. Neoplastic mast cell quantification in the supernatant. Sample H05-16.

Date	Days of cultivation	Passage	Nº of live cells in the supernatant/ml	Nº of dead cells in the supernatant/ml	Total (live and death) cells/ml	Percentage of viable cells
20/01/2016	0	P0	690000	60000	750000	92.0%
29/01/2016	9	P1	285000	160000	445000	64.0%
04/02/2016	15	P2	140000	180000	320000	43.7%
12/02/2016	23	P3	75000	115000	190000	39.4%
19/02/2016	30	P4	56000	133000	189000	29.6%
26/02/2016	37	P5	30000	143000	173000	17.3%
06/03/2016	46	P6	25000	192000	217000	11.5%
04/04/2016	74	P7	0	*	*	0.0%

Table S3. Neoplastic mast cell quantification in the supernatant. Sample H06-16.

Date	Days of cultivation	Passage	Nº of live cells in the supernatant/ml	Nº of dead cells in the supernatant/ml	Total (live and death) cells/ml	Percentage of viable cells
20/01/2016	0	P0	580000	30000	610000	95.1%
29/01/2016	9	P1	290000	105000	395000	73.4%
04/02/2016	15	P2	110000	155000	265000	41.5%
12/02/2016	23	P3	87000	220000	307000	28.3%
19/02/2016	30	P4	50000	255000	305000	16.4%
26/02/2016	37	P5	20000	160000	180000	11.1%
06/03/2016	46	P6	10000	140000	150000	6.7%
04/04/2016	74	P7	0	*	*	0.0%

Table S4. Neoplastic mast cell quantification in the supernatant. Sample H346-16.

Date	Days of cultivation	Passage	Nº of live cells in the supernatant/ml	Nº of dead cells in the supernatant/ml	Total (live and death) cells/ml	Percentage of viable cells
19/07/2016	0	P0	2800000	150000	2950000	94.9%
26/07/2016	7	P1	1100000	450000	1550000	71.0%
06/08/2016	18	P2	850000	1200000	2050000	41.5%
13/08/2016	25	P3	560000	1600000	2160000	25.9%
22/08/2016	34	P4	250000	800000	1050000	23.8%
12/09/2016	55	P5	180000	900000	1080000	16.7%
21/09/2016	64	P6	100000	900000	1000000	10.0%
28/09/2016	71	P7	0	*	*	0.0%

Table S5. Neoplastic mast cell quantification in the supernatant. Sample H19-18.

Date	Days of cultivation	Passage	Nº of live cells in the supernatant/ml	Nº of dead cells in the supernatant/ml	Total (live and death) cells/ml	Percentage of viable cells
06/02/2018	0	P0	2130000	27000	2157000	98.7%
14/02/2018	8	P1	1900000	180000	2080000	91.3%
23/02/2018	17	P2	1400000	780000	2180000	64.2%
06/03/2018	30	P3	950000	990000	1940000	49.0%
16/03/2018	40	P4	610000	1070000	1680000	36.3%
27/03/2018	51	P5	380000	1250000	1630000	23.3%
17/04/2018	61	P6	85000	1300000	1385000	6.1%
01/05/2018	75	P7	0	*	*	0.0%

Table S6. Neoplastic mast cell quantification in the supernatant. Sample H1171-18.

Date	Days of cultivation	Passage	Nº of live cells in the supernatant/ml	Nº of dead cells in the supernatant/ml	Total (live and death) cells/ml	Percentage of viable cells
22/08/2018	0	P0	775000	26000	801000	96.7%
31/08/2018	9	P1	615000	280000	895000	68.7%
10/09/2018	19	P2	440000	370000	810000	54.3%
18/09/2018	27	P3	195000	405000	600000	32.5%
27/09/2018	36	P4	125000	435000	560000	22.3%
13/10/2018	52	P5	45000	415000	460000	9.8%
24/10/2018	63	P6	0	*	*	0.0%

Table S7. Neoplastic mast cell quantification in the supernatant. Sample H1172-18.

Date	Days of cultivation	Passage	Nº of live cells in the supernatant/ml	Nº of dead cells in the supernatant/ml	Total (live and death) cells/ml	Percentage of viable cells
22/08/2018	0	P0	550000	78000	628000	87.6%
31/08/2018	9	P1	480000	140000	620000	77.4%
10/09/2018	19	P2	370000	395000	765000	48.4%
18/09/2018	27	P3	230000	480000	710000	32.4%
27/09/2018	36	P4	125000	520000	645000	19.4%
13/10/2018	52	P5	40000	435000	475000	8.4%
24/10/2018	63	P6	0	*	*	0.0%

Table S8. Neoplastic mast cell quantification in the supernatant. Sample H1249-18.

Date	Days of cultivation	Passage	Nº of live cells in the supernatant/ml	Nº of dead cells in the supernatant/ml	Total (live and death) cells/ml	Percentage of viable cells
01/09/2018	0	P0	800000	90000	890000	89.9%
14/09/2018	14	P1	610000	290000	900000	67.7%
24/09/2018	24	P2	420000	550000	970000	43.3%
03/10/2018	33	P3	310000	580000	890000	43.8%
13/10/2018	43	P4	115000	680000	795000	14.6%
22/10/2018	52	P5	50000	890000	940000	5.3%
03/11/2018	64	P6	0	*	*	0.0%

Table S9. Neoplastic mast cell quantification in the supernatant. Sample H232-16.

Date	Days of cultivation	Passage	Nº of live cells in the supernatant/ml	Nº of dead cells in the supernatant/ml	Total (live and death) cells/ml	Percentage of viable cells
30/04/2016	0	P0	1100000	85000	1185000	92.8%
06/05/2016	6	P1	630000	360000	990000	63.6%
16/05/2016	16	P2	330000	510000	840000	39.3%
24/05/2016	24	P3	87000	720000	807000	10.8%
30/05/2016	30	P4	57500	750000	807500	7.1%
06/06/2016	36	P5	0	*	*	0.0%

Table S10. Neoplastic mast cell quantification in the supernatant. Sample H334-16.

Date	Days of cultivation	Passage	Nº of live cells in the supernatant/ml	Nº of dead cells in the supernatant/ml	Total (live and death) cells/ml	Percentage of viable cells
24/06/2016	0	P0	890000	70000	960000	92.7%
04/07/2016	12	P1	565000	150000	715000	79.0%
09/07/2016	17	P2	470000	425000	895000	52.5%
18/07/2016	26	P3	205000	550000	755000	27.1%
26/07/2016	34	P4	125000	635000	760000	16.4%
04/08/2016	42	P5	0	*	*	0.0%

Table S11. Neoplastic mast cell quantification in the supernatant. Sample H395-17.

Date	Days of cultivation	Passage	Nº of live cells in the supernatant/ml	Nº of dead cells in the supernatant/ml	Total (live and death) cells/ml	Percentage of viable cells
11/05/2017	0	P0	1800000	5000	1805000	99.7%
20/05/2017	9	P1	1460000	505000	1965000	74.3%
29/05/2017	18	P2	980000	840000	1820000	53.8%
09/06/2017	29	P3	746000	786000	1532000	48.7%
18/06/2017	38	P4	478000	950000	1428000	33.5%
23/06/2017	43	P5	150000	800000	950000	15.8%
30/06/2017	50	P6	0	*	*	0.0%

Table S12. Neoplastic mast cell quantification in the supernatant. Sample H550-17.

Date	Days of cultivation	Passage	Nº of live cells in the supernatant/ml	Nº of dead cells in the supernatant/ml	Total (live and death) cells/ml	Percentage of viable cells
10/07/2017	0	P0	1700000	10500	1710500	99.4%
17/07/2017	7	P1	985000	690000	1675000	58.80%
22/07/2017	12	P2	420000	1040000	1460000	28.7%
01/08/2017	22	P3	126000	1370000	1496000	8.4%
09/08/2017	30	P4	0	*	*	0.00%

Table S13. Neoplastic mast cell quantification in the supernatant. Sample H603-17.

Date	Days of cultivation	Passage	Nº of live cells in the supernatant/ml	Nº of dead cells in the supernatant/ml	Total (live and death) cells/ml	Percentage of viable cells
19/10/2017	0	P0	3200000	245000	3445000	92.9%
30/10/2017	11	P1	1750000	1096000	2846000	61.5%
10/11/2017	22	P2	960000	1230000	2190000	43.80%
18/11/2017	30	P3	365000	1130000	1495000	24.40%
24/11/2017	36	P4	0	*	*	0.00%

Table S14. Neoplastic mast cell quantification in the supernatant. Sample H816-18.

Date	Days of cultivation	Passage	Nº of live cells in the supernatant/ml	Nº of dead cells in the supernatant/ml	Total (live and death) cells/ml	Percentage of viable cells
21/07/2018	0	P0	1800000	25000	1825000	98.6%
31/07/2018	10	P1	1020000	149500	1169500	87.2%
10/08/2018	21	P2	620000	350000	970000	63.9%
20/08/2018	31	P3	415000	615000	1030000	40.3%
27/08/2018	38	P4	120000	760000	880000	13.6%
03/09/2018	45	P5	0	*	*	0.0%

FIGURES

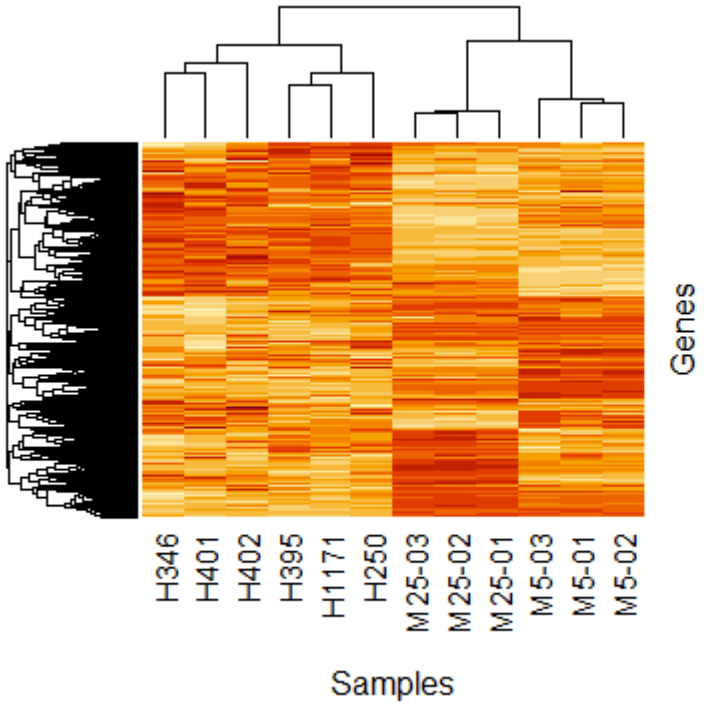

Figure S1. Hierarchical clustering heatmap of all differentially expressed genes between 6 canine mast cell tumour-derived fibroblasts (H346, H401, H402, H395, H1171 and H250) and two canine cancer cell lines (M5 and M25) demonstrating the clustering difference based on their molecular profile.

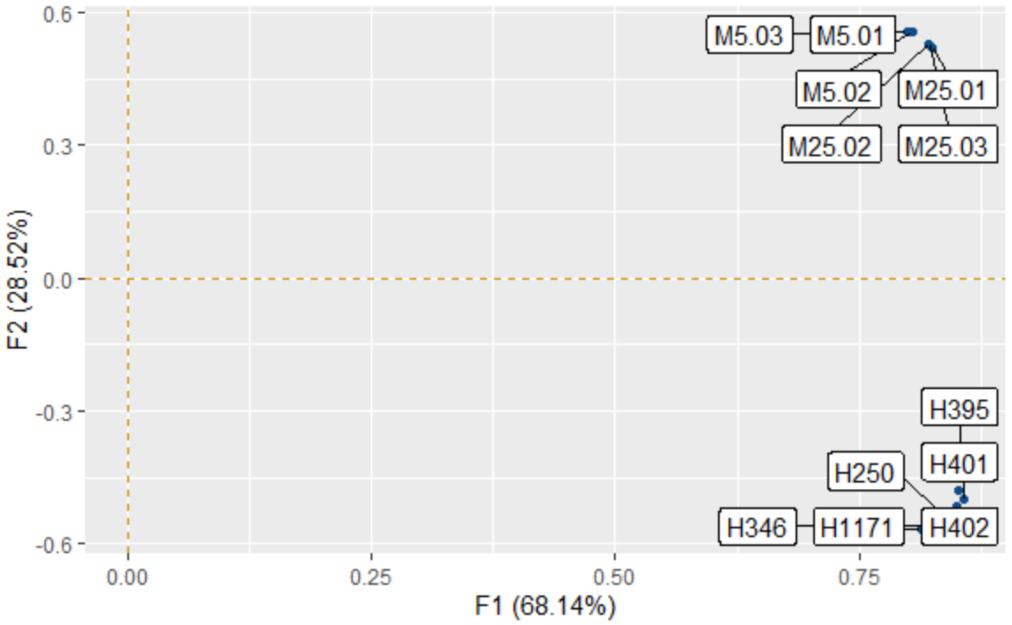

Figure S2. Perceptual Map from a PCA analysis based on the molecular profile of mast cell tumour-derived fibroblasts and canine cancer cells. First two principal components are shown exhibiting a clear separation between the fibroblasts and canine cancer cells based on their molecular profile.
